# Synthesis and characterization of ceramic refractories based on industrial wastes

**DOI:** 10.1038/s41598-024-74997-y

**Published:** 2024-10-24

**Authors:** G. A. Khater, M. Romero, A. López-Delgado, I. Padilla, A. A. El-Kheshen, M. M. Farag, M. S. Elmaghraby, N. H. S. Nasralla

**Affiliations:** 1 Glass Research Department, National Research Centre, Dokki, Cairo Egypt; 2 Eduardo Torroja Institute for Construction Sciences, IETcc-CSIC, Madrid, Spain; 3 Refractories,Ceramics and Building Materials Department, National Research Centre, Dokki, Cairo Egypt; 4 Electron Microscopy and Thin Films Department, National Research Centre, Dokki, Cairo Egypt

**Keywords:** Sintering, Porosity, Density, Cordierite, Mullite, Ceramic roller, Environmental sciences, Materials science

## Abstract

The possibility of reusing ceramic roller waste to produce cordierite and mullite refractories was investigated. Five batches were designed using wastes representing ceramic roller waste, magnesite, and silica sand, shaped and fired at 1300 °C/2 h, and one batch was selected at 1200 °C. The chemical composition and precipitated phases of the used raw materials and the fired batches were analyzed using XRF and XRD techniques, respectively. Densification parameters, morphology, microstructure and electrical properties were also studied to evaluate the effect of the formed phases on the properties of fired materials. Bulk density increases with an increase in mullite and a decrease in cordierite, and it also increases with increasing temperature, whereas porosity and water absorption show a opposite behavior to density (it decreases with an increase in mullite and temperature). The main phases developed after firing at 1300 °C/2 h were cordierite, mullite, corundum, baddeleyite, and spinel. Bending strength increases with increasing mullite percentage and density, and decreasing grain size and porosity. The microstructure develops and becomes finer with increasing mullite percentage and density. The grain size of the crystals was very fine at 1200 °C/2 h and increased at 1300 °C/2 h. Broadband dielectric spectroscopy was employed to study the electrical and dielectric behavior of the investigated samples. The increase in mullite concentration shows a remarkable increase in ε’, especially at lower frequencies, as it is three times higher than that of M10. At f > 103 Hz ε’, frequency independence is accompanied by an increase in mullite concentrations due to the lag of dynamics fluctuations after the alteration of the electric field. The generation of new free ions leads to the enhancement of conductivity as the mullite concentration increases.

## Introduction

Over the past 100 years, there has been an exponential increase in the volume of industrial waste. Every year, more than 25 billion tonnes of solely solid technology waste are created worldwide. Technogenic waste and geoecological issues are closely related to regional economic development and environmental conservation. The production of waste is another sign that natural resources are being used unsustainably when many are almost depleted. Consequently, recycling industrial waste is a crucial duty for the environment, economy, geoecology, and natural resources. It involves the development of structural and functional materials with excellent performance qualities and the extraction of valuable and deficient materials from industrial waste, such as radioactive, pure oxides, non-ferrous, noble, rare, and other elements^1,2^. Khater et al.^3–7^ used many industrial wastes to produce lightweight, highly porous ceramic materials and glass-ceramic materials with good mechanical properties that qualify them for use in building materials.

Refractory materials are brittle, inorganic, and crystalline solids burned at the ideal temperature for their intended use. They serve as thermal insulation on the front and rear of industrial furnaces at high temperatures. They are categorized into acidic, neutral, and basic refractories. This classification is based on their chemical composition and resistance to being corroded by the burned materials and in contact with the refractory material in a firing environment. Aluminosilicate refractories rich in silica are considered acidic refractories, whereas those rich in alumina belong to the neutral type^8–11^.

Refractories experience mechanical abrasion at high temperatures, corrosion from liquids and gases, and different degrees of mechanical stress and strain. They are intended to be heat resistant. The performance of a refractory, which includes its ability to withstand heat shock and maintain its integrity, is closely linked to its texture and the abundance of refractory phases, including mullite, corundum, periclase, doloma, spinel, and alumina^12,13^.

Special landfills are used for the final disposal of waste materials, which are also a significant source of secondary raw materials for the fabrication of refractories. The biggest obstacle to reducing reliance on primary raw material imports and worldwide CO_2_ emissions is the implementation of a comprehensive industrial recycling procedure for refractory materials^14^.

Massive growing amounts of ceramic waste in the form of ceramic sludge, ceramic roller, and broken tiles are produced during the ceramic fabrication process, causing moderate to severe environmental problems. Another problem in the operation of ceramic production furnaces is the spent rollers. These are usually fabricated from high alumina, silica, and zirconia—the monthly product of the ceramic roller waste accumulating within the plant premises as stockpiles^15,16^.

One of the most significant phases of the MgO–SiO_2_–Al_2_O_3_ system is cordierite (2MgO–2Al_2_O_3_–5SiO_2_), which has a very low thermal expansion coefficient and good resistance to thermal shock. It also exhibits excellent mechanical strength, good refractoriness, great mechanical stability, low dielectric constant, and high volume resistivity. Accordingly, cordierite is utilized as a refractory material, substrate material for integrated circuit boards, honeycomb-shaped catalyst carrier in automotive exhaust systems, and as an alternative to alumina in electronics. Most cordierite in nature comprises Al_3_(Mg, Fe)_2_Si_5_AlO_18_. It exhibits orthorhombic crystals and low-temperature polymorphism, although its hexagonal form is observed at high temperatures. Khater et al.^17^ produced high-performance cordierite and wollastonite ceramics from industrial wastes presented in arc and blast furnace.

Cordierite (M_2_A_2_S_5_) has a stoichiometric composition of 13.7 mass% MgO, 34.9 mass% Al_2_O_3_, and 51.4 mass% SiO_2_. Iron cordierite melts incongruently at 1210 °C to form mullite (3Al_2_O_3_·2SiO_2_), tridymite (SiO_2_), and liquid phase, while magnesium cordierite melts incongruently at 1465 °C to form mullite^18–25^.

The single stable crystalline phase in the Al_2_O_3_–SiO_2_ system is mullite, which has enormous significance in both conventional and highly developed ceramics. This is principally because of its properties: low thermal expansion, high resistance to creep, high thermal stability, conductivity, and corrosion resistance. Its fracture toughness and mechanical strength are also good. Mullite is the crystalline phase developed by heating clay materials^26–28^.

The scope of the present research is to study the possibility of reusing ceramic roller waste and magnesite to produce cheap ceramic refractories containing cordierite and mullite phases with little or no secondary crystalline phases and to protect and to clean the environment from these harmful wastes. Also the prepared materials in this work can be used as electrical insulating ceramic materials beside their usage as refractory materials. such as ceramic arc tubes, protective parts, and spark plugs^29^.

## Experimental techniques

### Batch calculation and sample preparation

**Fig. 1:**
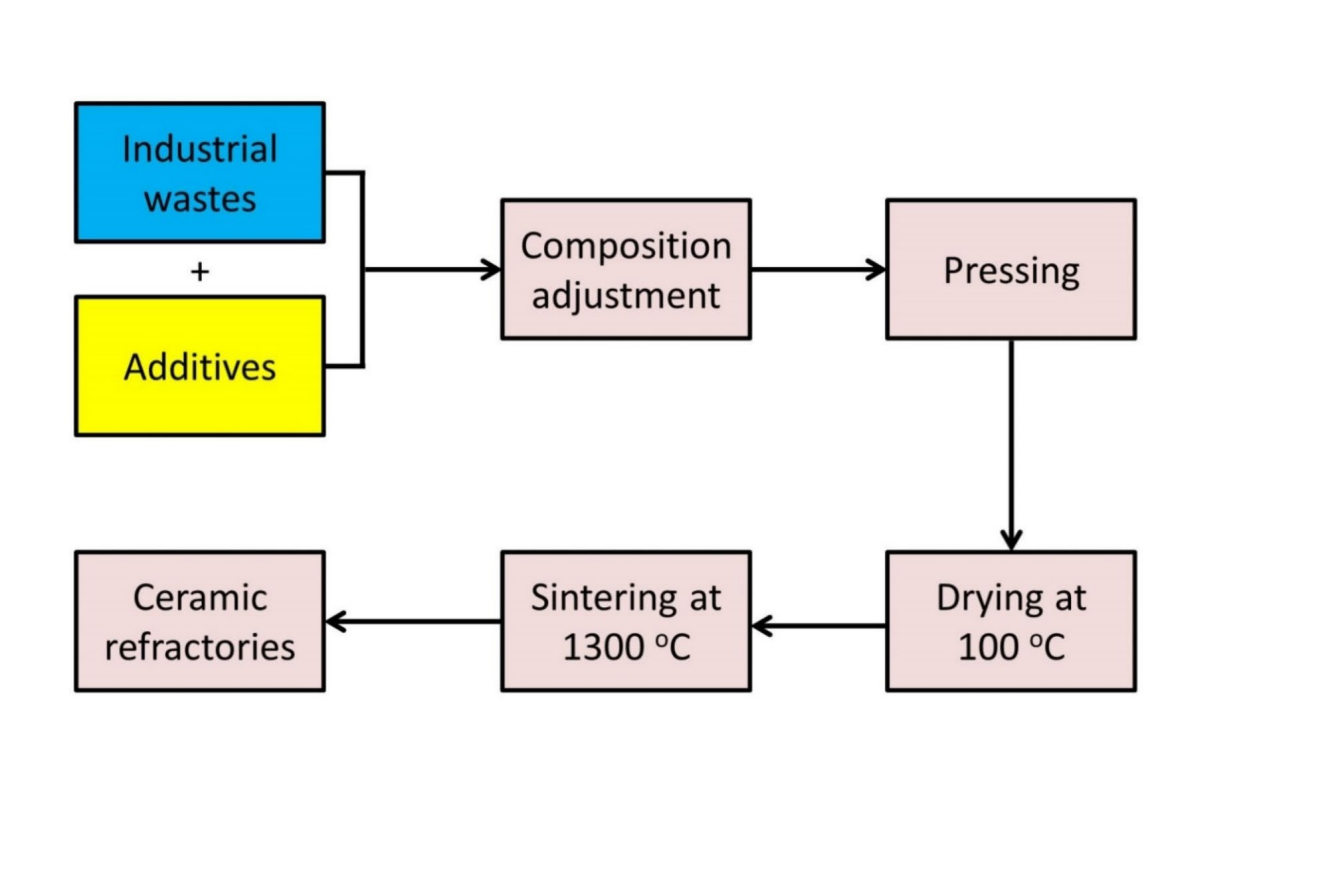
Schematic procedure for the production of ceramic refractories from industrial waste.

Mullite-cordierite based refractory materials were fabricated, from ceramic roller waste, magnesite minerals, and silica sand, following the procedure shown in Fig. 1. The ceramic roller waste was supplied by Ceramica Venezia Company (6th of October City, Egypt), while the magnesite and silica sand were received from El-Nile mining company (Egypt). All raw materials were crushed and ground in a ball mill for 1 h until homogeneous, and then passed through a 63-micron sieve. The chemical composition of raw materials was analyzed by X-ray Fluorescence (XRF) (model pw/ 2404). The percentage of oxides was determined using SuperQ and SemiQ software with an accuracy of 99.99% and a confidence limit of 96.7%. Table 1 collects the XRF results.

**Table 1:** Chemical composition (XRF, expressed as wt% oxide) and loss of ignition (LOI) (%) of the ceramic roller waste, magnesite and silica sand.

Oxide	Ceramic roller	Magnesite	Silica sand
SiO_2_	28.19	2.85	98.35
Al_2_O_3_	60.00	1.94	1.24
Fe_2_O_3_^tot^.	0.76	1.48	0.26
TiO_2_	0.52	-	trace
MgO	0.48	36.92	trace
CaO	0.43	8.56	0.10
ZrO_2_	6.95	-	-
BaO	0.39	-	-
P_2_O_5_	0.32	-	-
Na_2_O	0.69	-	trace
K_2_O	0.68	-	trace
L.O.I.	-	48.14	0. 39

Five mixtures (M10, M20, M30, M40, and M50), presented in Table 2, were prepared from the ceramic roller waste, magnesite and silica sand, based on different nominal percentages of mullite and cordierite in the fired refractory materials. The powdered raw materials were weighed and well mixed to the proper ratio. Approximately 5% H_2_O was added to 5 g of each sample as a binder and pressed in a cylindrical mold of about 50 mm diameter and 50 mm depth using a uniaxial weight of 40 MPa. The shaped samples were dried in an oven furnace at approximately 120 °C for 20 h and then treated at 1300 °C for 2 h in a single stage. The sample M30 was chosen to study the effect of sintering temperature on crystalline phases development on firing. To this end, M30 sample was also sintered at 1200 °C for 2 h.

**Table 2:** Designation of the different mixtures studied, as well as the percentage of raw materials and the content of silicon, aluminium and magnesium oxides in each mixture.

Batch no.	Nominal phase		Batch composition (wt%)			Batch constituents (wt%)		
	mullite	cordierite	SiO_2_	Al_2_O_3_	MgO	Ceramic roller	Magnesite	Silica sand
M 10	10	90	49.04	38.56	12.40	49.62	26.37	24.01
M 20	20	80	46.73	42.25	11.02	55.18	23.72	21.10
M 30	30	70	44.41	45.94	9.65	60.87	21.01	18.12
M 40	40	60	42.10	49.64	8.27	66.72	18.22	15.06
M 50	50	50	39.76	53.35	6.89	72.72	15.36	11.92

The mineralogical characterization of the raw materials and refractories was performed by X-ray diffraction (XRD) using a Bruker equipment (D8 Discover) with CuKα radiation, with 2θ from 10° to 80°. The microstructure of the obtained refractories was examined by Field Emission Scanning Electron Microscopy (FESEM) in a Quanta (250 FEG) microscope equipped with an energy dispersive X-ray spectroscopy detector (EDS), and using an acceleration voltage of 30 kV.

The water absorption, A (%), of the fired specimens was calculated using this equation.

$$\:A\%=\frac{Mu-Ms}{Ms}x100$$

where Mu is the sample mass (g) after immersion in water for 24 h and Ms is the sample mass before (g) water immersion.

The bulk density, BD (g/cm^3^) is the weight of 1 cm^3^ of a bulk refractory test piece and it is calculated according to the following equation:

BD = [w_1_ / (w_2_ – w_3_)] x ρ _liquid_.

where w1 is the weight (g)of the dry tested sample, w_2_ is the weight (g) of the saturated tested sample in air, w_3_ is the weight (g) of the saturated test piece immersed in the liquid, and ρ (g/cm^3^) is the specific weight of the used liquid.

The apparent porosity, AP (%), is the percentage of open-pore volume relative to the volume of the test piece and is calculated according to the following equation:

AP = [(w_2_ - w_1_) /( (w_2_ - w_3_)] x 100.

The three-point bending method was used to test the bending strength of the prepared refractory samples, with dimensions of height, width, and length equal to 10, 12, and 50 mm, respectively. The samples were tested using a universal testing machine (model Tinius Olsen 10ST) with a span of 30 mm and loading speed of 0.5 mm/min.

Finally, a high-resolution Alpha analyzer, a Novocontrol Concept 40 instrument, was used to examine the electrical conductivity and dielectric characteristics in the frequency range of 0.1 Hz to 20 MHz. All measurements were carried out at room temperature with an accuracy greater than 99%. The samples were prepared between two gold-plated stainless-steel electrodes in a parallel plate capacitor configuration. More details about the setup can be found elsewhere^30^.

## Results and discussion

### Chemical and phase compositions of raw materials

**Fig. 2:**
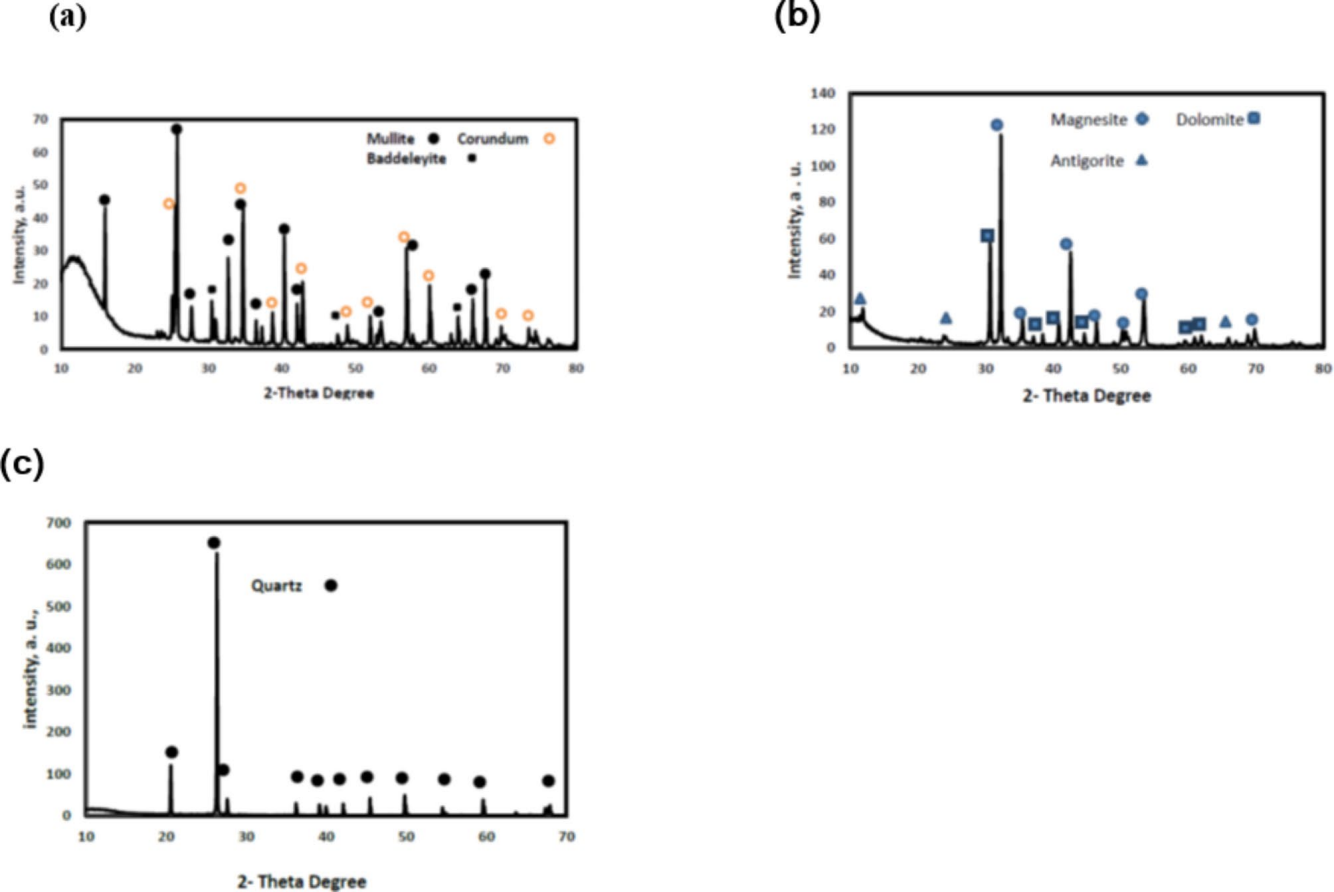
(**a**) XRD pattern of the ceramic roller waste, (**b**) XRD pattern of the magnesite, (**c**) XRD pattern of the silica sand.

Table 1 collects the chemical composition of the ceramic roller, magnesite, and silica sand and Fig. 2 shows the X-ray diffraction of all raw materials. The ceramic roller is essentially composed of alumina and silica in addition to zirconia, together with minor and trace amounts of other oxides. It is composed of mullite, corundum, and baddeleyite (ZrO_2_) (Fig. 2a), which is in accordance with its chemical composition.

Magnesium and calcium oxides are the primary oxide content in magnesite raw material, together with silica, alumina, and iron oxides in a minor extent. The loss on ignition is 48.14% and is likely related to the release of carbon dioxide and water due to the decomposition of the constituent phases. Indeed, the mineralogical composition of the magnesite sample (Fig. 2b) supports its chemical composition as magnesite (MgCO_3_) is the primary phase together with dolomite (CaMgCO_3_) and antigorite Mg_3_Si_2_O_5_ (OH)_4_ as minor components.

The chemical composition of the silica sand sample mainly contains silica 98.35% with a minor amount of alumina and iron oxide. Concernig its mineralogical composition (Fig. 2c), quartz is the only crystalline phase detected in its X-ray diffractogram, confirming its chemical composition consisting mainly of silica.

**Fig. 3:**
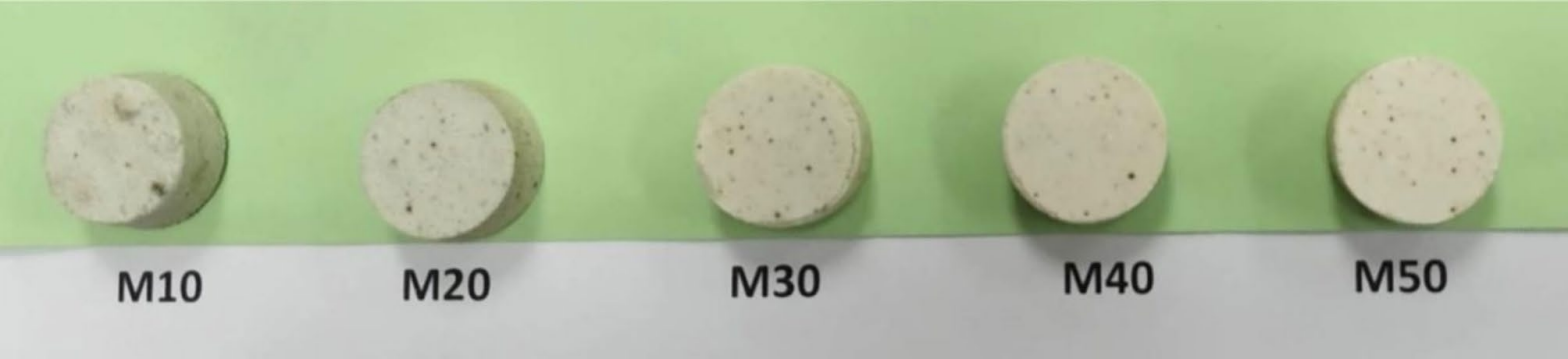
Appearance of the investigated refractories after sintering at 1300 ^o^C for 2 h.

Table 2 shows the designation of the different mixtures studied, as well as the percentage of raw materials and the content of silicon, aluminium and magnesium oxides in each mixture. These combinations of raw materials were calculated to yield different theoretical amounts of mullite and cordierite in the refractories. Once the specimens were formed, they were fired at 1300 °C for 2 h. Figure 3 shows the appearance of the materials resulting after firing.

### Physical properties of the fired samples

**Fig. 4:**
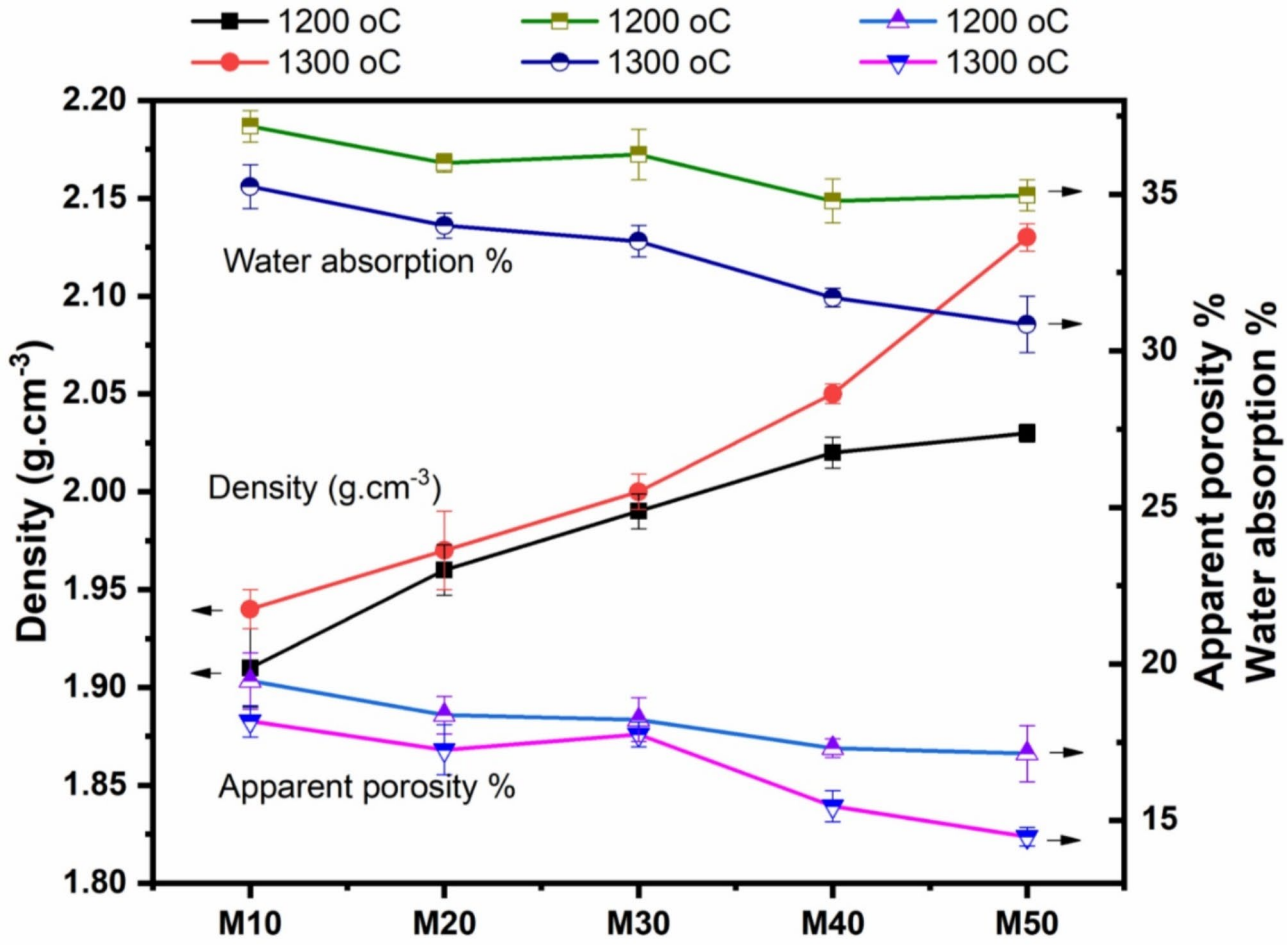
Density (g.cm^− 3^), apparent porosity (%), and water absorption (%) of samples fired at 1200 ºC and 1300 ºC for 2 h.

The physical properties of samples fired at 1200 ^o^C/2 h and 1300 ^o^C/2 h, specifically bulk density, apparent porosity, and water absorption, are presented in Tables 3 and 4; Fig. 4. When the samples are fired at 1200 ^o^C/2 h (Table 3f; Fig. 4a), the density increases from M10 to M50, ranging from 1.91 to 2.03 g/cm^3^. In contrast, porosity and water absorption decreases, ranging from 37.17 to 34.79, and 19.46 to 17.14, respectively. As the temperature increases up to 1300^o^C, the density further increases, while the porosity and water absorption continue to decrease (Table 4; Fig. 4b). The increase in density from M10 to M50 is attributed to the higher percentage of mullite, which has a density of 3.16 g/cm^[331^, and the corresponding decrease in cordierite, which has a density of 2.30 g/cm^[332^. The high density influences the pores, reducing the number of voids that affect the water absorption rate. When the temperature is raised to 1300 ^o^C, a glassy phase forms, leading to an increase in density and a decrease in both porosity and water absorption. These findings are consistents with other studies, which have reported that the densities of mullite, corundum, and spinel phases^33^, are higher than that of cordierite^34^. Specifically, the densities of spinel and corundum in those studies are 3.58 and 3.97 g/cm^3^, respectively, while that of cordierite is 2.3 g/cm^3^. These prior data are align with the results of the current study. Mouiya et al.^35^ indicated that the increase in bulk density caused by firing is likely due to forming a glassy phase, resulting in more significant liquid phase production and reduced porosity.

**Table 3:** Densification parameters of samples fired at 1200 ^o^C for 2 h.

Batch no.	Densification parameters		
	Bulk density (g/cm^3^)	Apparent porosity (%)	Water absorption (%)
M10	1.91	37.17	19.46
M20	1.96	36.00	18.37
M30	1.99	36.27	18.22
M40	2.02	34.97	17.31
M50	2.03	34.79	17.14

**Table 4:** Densification parameters of samples fired at 1300 ^o^C for 2 h.

Batch no.	Densification parameters		
	Bulk density (g/cm^3^)	Apparent porosity (%)	Water absorption (%)
M10	1.94	35.25	18.17
M20	1.97	34.00	17.26
M30	2.00	33.50	16.75
M40	2.05	31.70	15.46
M50	2.13	30.84	14.48

###  X-ray diffraction studies of the sintered samples

#### X-ray diffraction of all samples

**Fig. 5:**
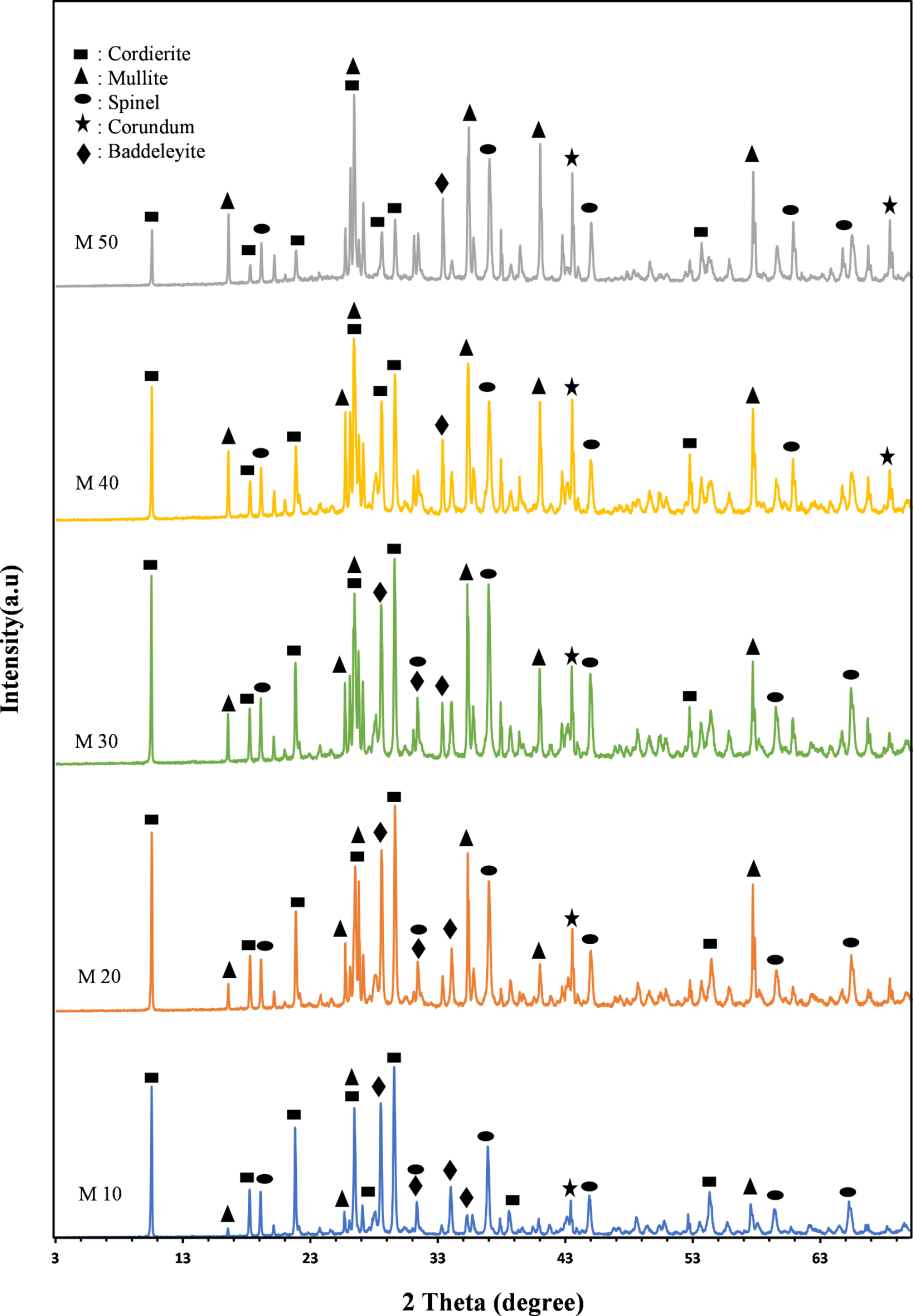
X-ray patterns of the samples after firing at 1300 ^º^C for 2 h.

Figure 5 shows the XRD patterns of the studied compositions fired at 1300 °C for 2 h. Cordierite ((MgFe)_2_Al_4_Si_5_O_18_) (PDF 013–0294) is the main phase formed in samples M10 to M30, with characteristic lines at 8.58, 3.38, 3.03, 3.18, and 4.11 Å, and it slightly decreases in samples M40 and M50. This reduction occurs at the expense of mullite (Al_6_Si_2_O_13_) (PDF.06-0258), which constitutes the second phase and is characterized by lines at 3.39, 3.43, 2.54 Å, as well as corundum (Al_2_O_3_) (PDF 01-073-5928), with lines at 2.08, 2.55 and 1.60 Å. Additionally, a spinel phase (MgAl_2_O_4_) (PDF 01-071-6331) is formed in all samples, with lines at 2.43, 2.85, and 1.43 Å, along with a small amount of baddeleyite (ZrO_2_) (PDF 013–0307), characterized by lines at 3.16 and 2.83 Å. and 2.62 Å. The mullite phase shows minimum content in batch M10 and increases progressively up to batch M50 as the ceramic roller percentage in the composition and magnesia decreases. The formation of spinel and corundum phases in all samples and their development from M10 to M50 is due to the low percentage of silica, which facilitates the reaction of magnesia with mullite to produce spinel and corundum. The evolution of baddeleyite from M10 to M50 is also attributed to the increase in mullite content and the a high proportion of zircon in the ceramic roller (Tables 1 and 2).

This results are consistent with Simon Sembiring et al.^36^, who studied the effect of adding Al_2_O_3_ on the conversion and crystallization behavior of cordierite refractory ceramics based on rice husk silica under heat treatment regime at approximately 1230 °C. Their results indicated that the addition of Al_2_O_3_ led to transformation of cordierite into spinel and corundum. Furthermore, they found that adding 10–30% Al_2_O_3_ increases the amount of spinel while reducing the amount of corundum and cristobalite phases. The occurrence of corundum, spinel, and cristobalite was associated with an increase in density and flexural strength, whereas a decrease in porosity was observed. After the addition ofapproximately 15–30% alumina, the coefficient of thermal expansion of the stabilized at a comparatively constant value of 9.5 × 10^− 6^/ºC, with the principal crystalline phase being spinel, followed by corundum and lesser quantities of cristobalite.

#### Effect of firing temperatures on crystalline phase formation

**Fig. 6:**
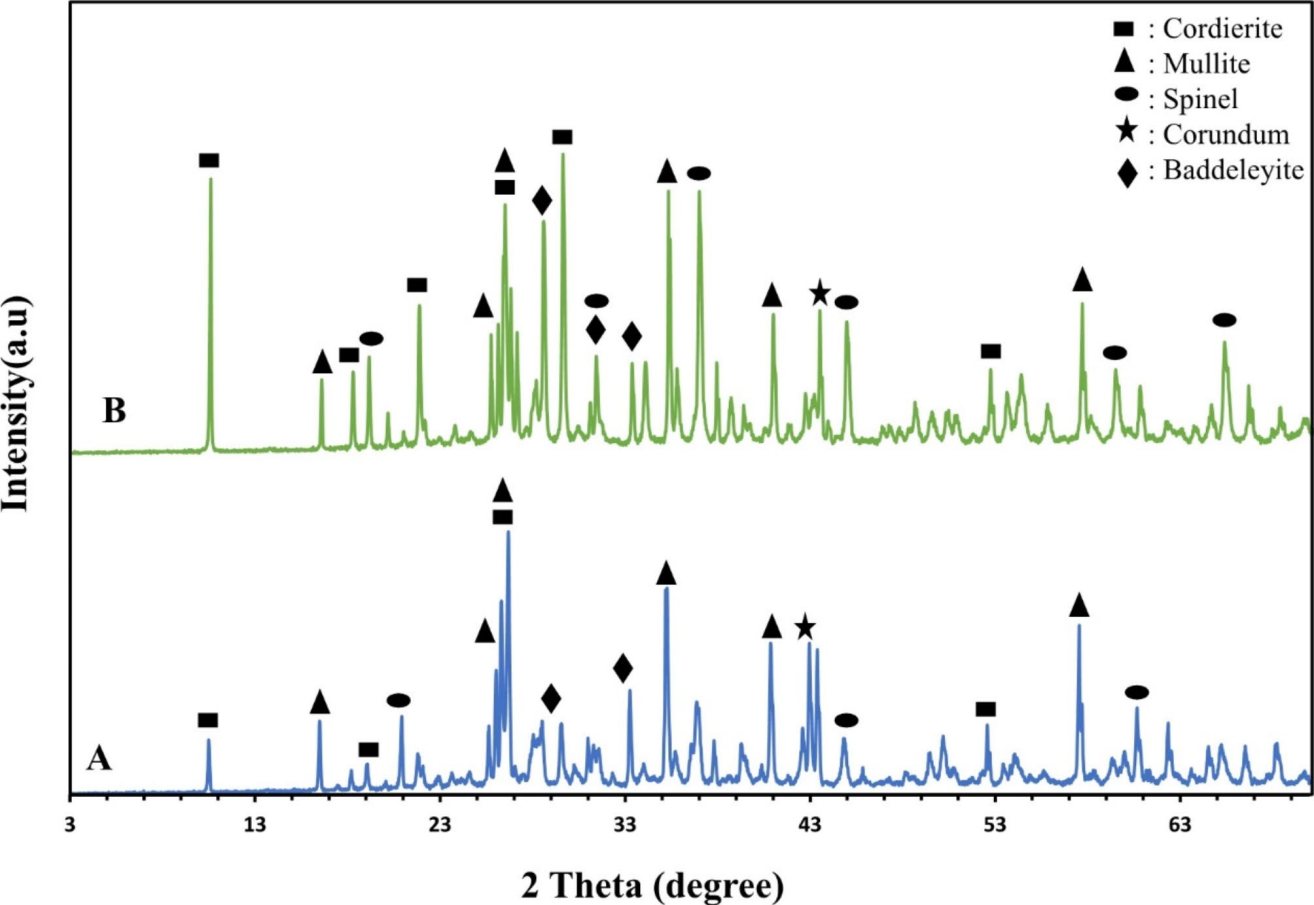
X-ray patterns of sample M30 after firing at different temperatures (A = Fired at1200 ^º^C/2 h, and B = Fired 1300 ^º^C/2 h.

The effect firing temperatures was investigated on sample M30 because it represents the median composition of those studied. Sample M30 was sintered at both 1200 º and 1300 °C for 2 h. Figure 6 shows the diffraction patterns of M30 after firing. At 1200 °C, the crystalline phases exhibit weak formation, indicated by the decreased intensity of their lines. Cordierite is the primary phase, followed by mullite and spinel phases, with corundum and baddeleyite being the least noticeable. When the temperature was increased to 1300^o^C, the crystalline phases are more developed, and the intensity of their diffraction lines increased for all crystalline phases. This suggests that at 1200 ^o^C, a significant proportion of the material remains in an amorphous glassy phase. As the temperature increases, the viscosity of the liquid phase decreases, allowing for the development of crystalline phases, thereby enhancing the intensity of the diffraction lines. These findings agree with the study by Cao, Zhimin, and Li, Ping^37^, who demonstrated that increasing the firing temperature led to a higher intensity intensity of the cordierite diffraction peak.

###  SEM and EDX analysis of the sintered samples

**Fig. 7:**
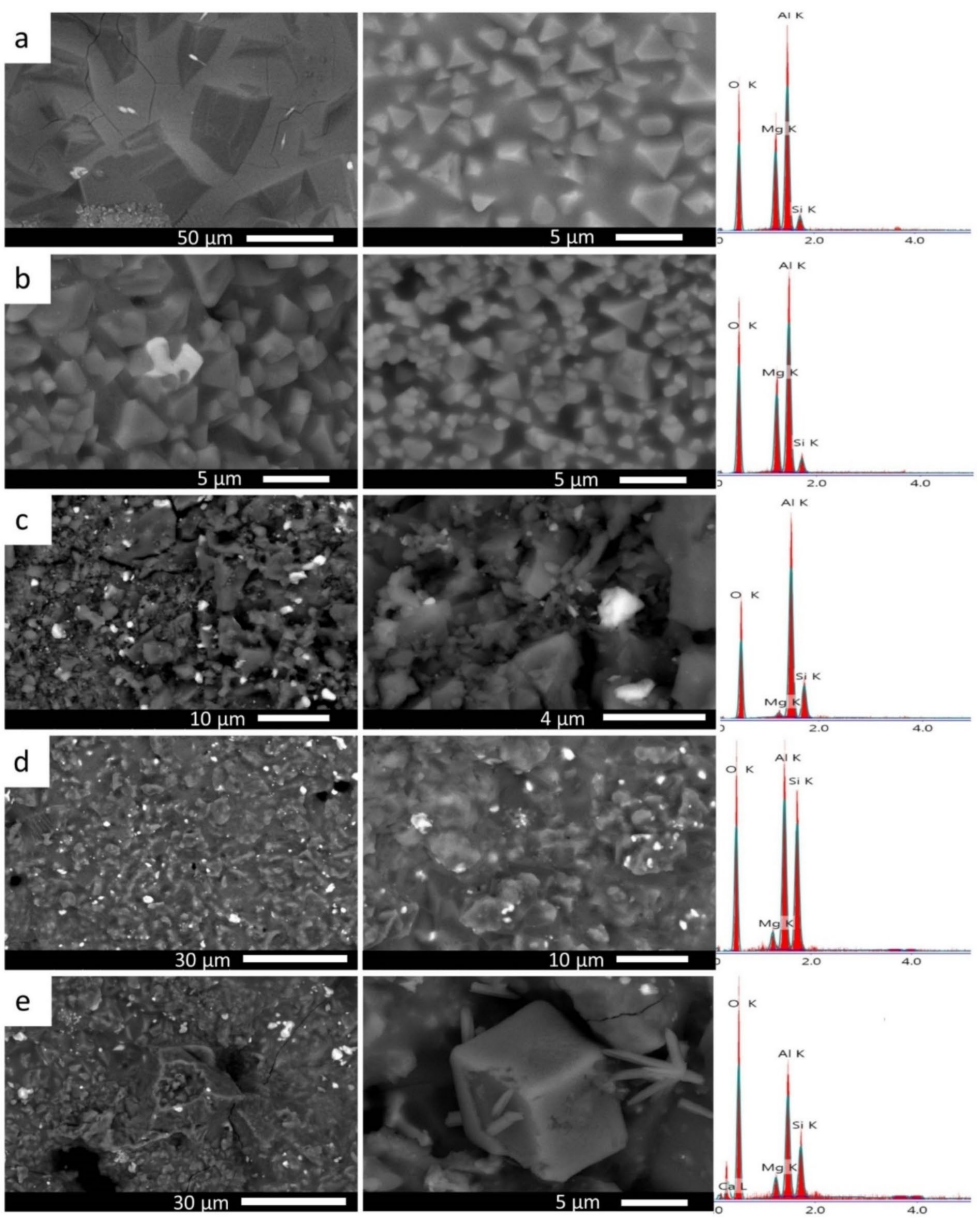
SEM images and EDS analysis of M10, M20, M30, M40, and M50 (**a-e**, respectively) samples fired at 1300 ^o^C for 2 h.

The microstructure of fired materials significantly influences their physical properties, such as density, porosity, making its study essential. Figure 7 shows the microstructure observed in M10, M20, M30, M40, and M50 samples. Their elemental surface composition of is collected in Table 5. In the M10 sample (Fig. 7a), the presence of euhedral to subhedral crystals of mullite and cordierite crystals is evident. Mullite is a remnant phase of the crystalline composition of the ceramic roller residue, while cordierite is a neo-formation phase formed through reactions between the components of the original raw materials (silica sand, magnesite, and ceramic roller)^38–40^. Furthermore, tiny grains of corundum embedded within the matrix are observed. EDS elemental analysis reveals the presence of Mg, Al, and Si, which confirmes the formation of the aforementioned crystalline phases.

**Table 5:** Elemental analysis (atomic %) of different samples carried out by EDS analysis.

Element	M10	M20	M30	M40	M50
O	55.32	58.84	62.14	65.6	73.76
Mg	14.01	12.79	1.08	2.03	1.99
Al	27.43	25.49	28.27	14.44	8.18
Si	3.24	2.88	8.51	13.24	3.81

For the M20 (Fig. 7b)sample, similar to M10, cordierite and mullite are present with coarse to refined grains of subhedral and euhedral crystals. Meanwhile, zircon appears as bright irregular crystals within mullite and cordierite crystals (EDS data not shown). As the ceramic roller content increases in the refractory composition, the microstructure becomes more densely compacted, with higher corundum content and a significant presence of mullite and a smaller amount of cordierite. The microstructure of sample M30 (Fig. 7c) features baddeleyite as bright white irregular crystals within a corundum-rich matrix. The M40 sample comprises irregular grains, predominantly of mullite with a lesser amount of cordierite. A high content of corundum is observed as a fine-grain matrix interspersed with roller fragments (Fig. 7d). Sample M50 (Fig. 7e) primarily consists of mullite and corundum with minor amounts of calcium silicate detected in M40 and M50. These appears fibrous and rod-like crystals within the mullite and cordierite matrix, confirmed by EDS analysis.

**Fig. 8:**
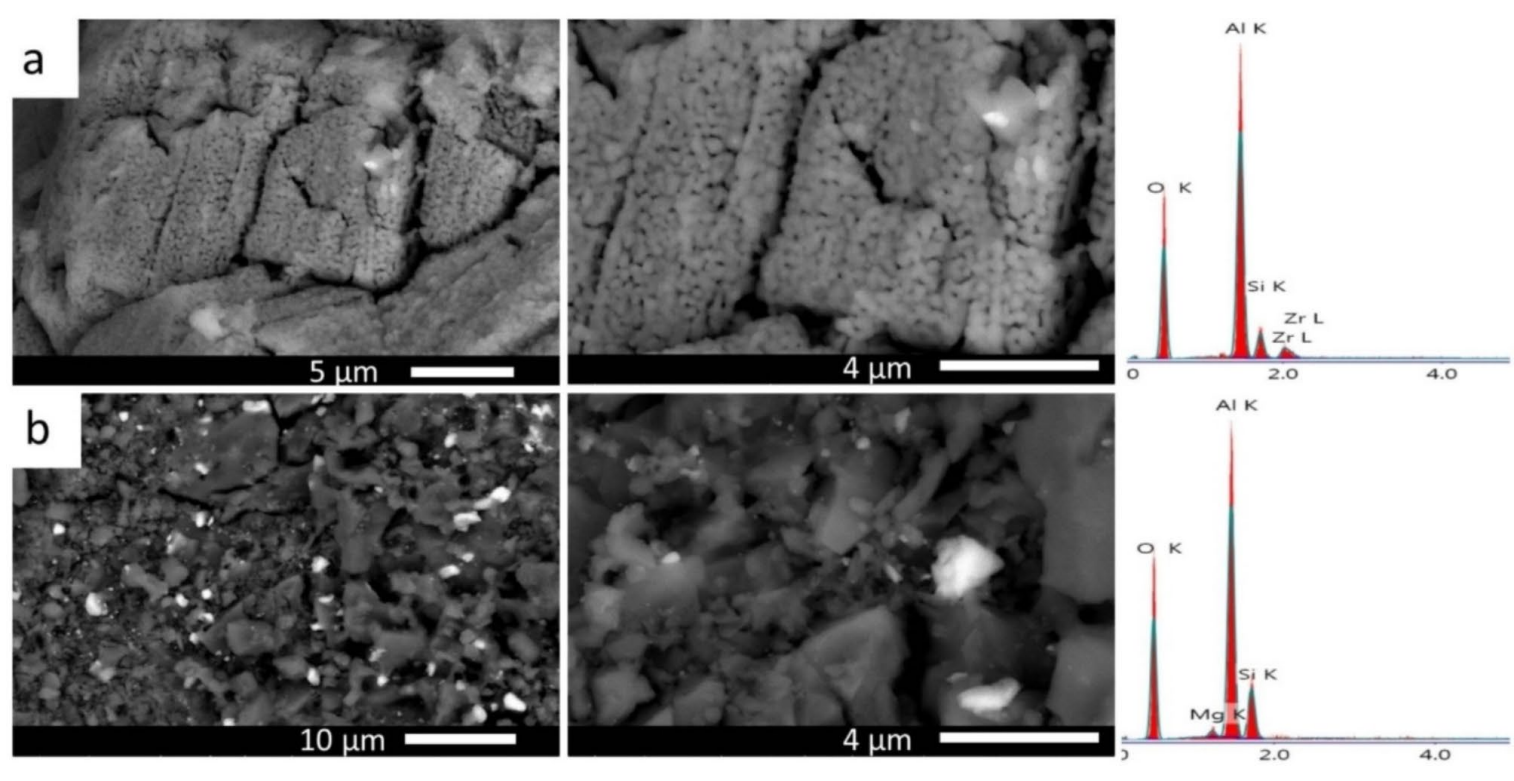
SEM images and EDS analysis of M30 fired at 1200 ^o^C/2 h (a) and 1300 ^o^C/2 h (b).

The effect of the firing temperature on the microstructure was specifically examined for sample M30, selected because it achieved optimum properties in this study. Figure 8 (a, b) shows FESEM images and EDS analysis of M30 sample fired at 1200 °C The crystal size is significantly coarser than that fired at 1300 ^o^C, due to the incomplete reaction of magnesia and silica inform the raw materials with the ceramic roller at low temperatures, which hinders further crystal growth.

### Bending strength (BS)

**Fig. 9:**
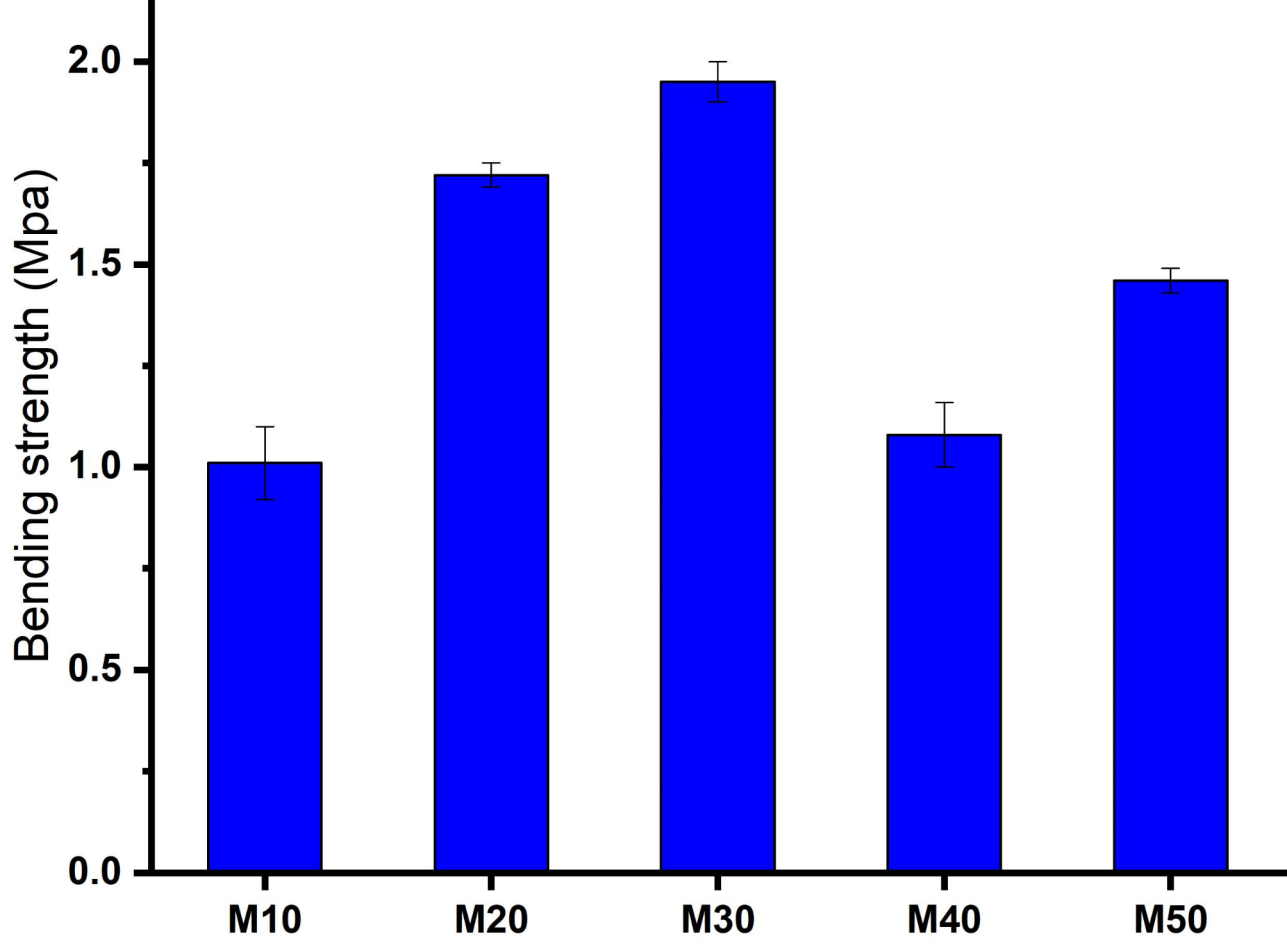
Bending strength of samples fired at 1300 ^o^C for 2 h.

Figure 9 illustrates the bending strength (MPa) of the M10, M20, M30, M40, and M50 samples. Bending strength increases with the mullite content up to 30% of mullite, reaching a maximum value of 1.95 MPa. Beyond this point, the bending strength decreases. The increase in bending strength from M10 to M30 is attributed to a decrease in porosity, as indicated in Table 3. Porosity leads to stress concentration and a reduction in the load-receiving area. This findings aligns with the research conducted by Simon et al.^40^ and Barsoum^41^.

###  Electrical and dielectric properties

The capability of a material to store electrical energy is determined by its permittivity, also referred as the dielectric constant (ε’). When charge carriers accumulate at metal electrodes or between different material phases, it induces polarizations that enhance the electrical capacity and permittivity of the condenser. For optimizing the electrical performance of energy storage devices, maximizing their permittivity to its utmost level is essential. The relationship between the real part of the dielectric constant (ε’) and electrical capacitance (C) can be expressed as follows^42^:

ε‘(ω) = C(ω)d/(ε_o_ A).

where ω is the angular frequency (ω = 2πf) with f being the applied electrical field frequency in Hertz, ε_o_ = 8.85 × 10^− 12^ F/m is the vacuum permittivity. The sample geometry is characterized by d (thickness) and A (sample surface area).

**Fig. 10:**
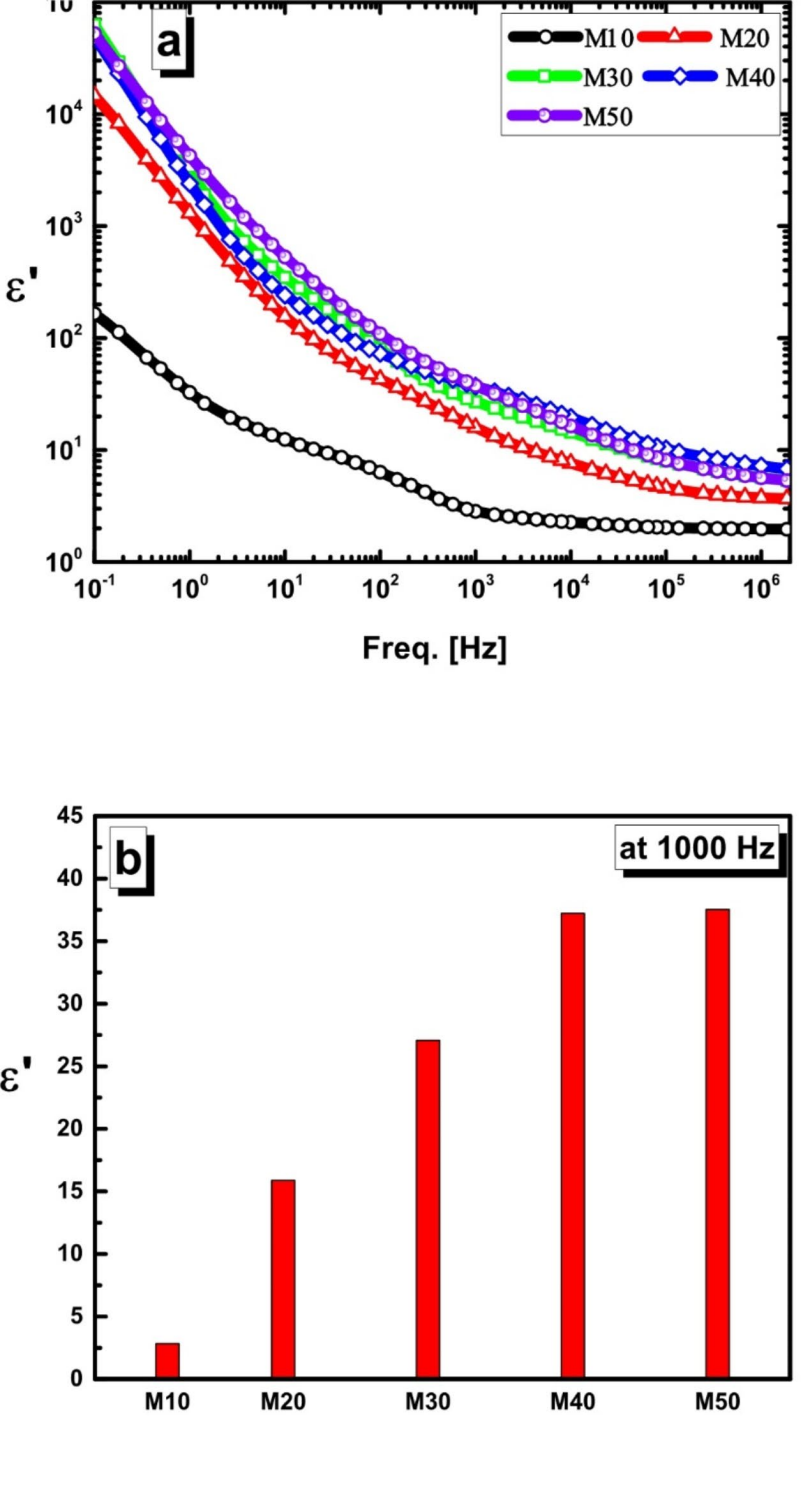
(**a**) Frequency dependence of permittivity (*ε’*) of the investigated samples, and (**b**) determined values of *ε’* at the representative frequency of 1 kHz for the different compositions.

The frequency dependence of the permittivity ε’ is shown in Fig. 10. Furthermore, Table 6 provides the calculated values of ε’ and AC-conductivity,σ′, for selected frequencies. Two distinct trends are observable. First, from 1 kHz to 0.1 Hz, the permittivity increases rapidly as the frequency decreases. This behavior is primarily attributed to the superposition of the interfacial polarization^43,44^, which is typically present in such heterogeneous materials, and the contribution from charge carrier transport, which results in increases conductivity. A significant increase in ε’ in the low-frequency domain (< 10^3^ Hz) becomes apparent with higher mullite concentrations, showing a rise by three orders of magnitude.

Table 6 shows a gradual decrease in permittivity, which becomes almost frequency-independent at frequencies above 10^3^ Hz. The permittivity values decrease, remained nearly constant, and and exhibit a significant drop one decade over the entire frequency range, indicating that neither composition nor frequency has a substantial effect on the permittivity values. This result can be explained by the fact that at higher frequencies, changes in polarizations or charge carrier hopping mechanisms lag behind the frequency of the applied electric field^45,46^ because of the applied field changes much rapidly than the dynamic response of the samples under study^47^. Consequently, as the concentration of mullite increases, ε’ becomes frequency-independent, showing less dependence on mullite concentrations.

**Table 6:** Permittivity, ε′ and AC-conductivity of the samples as determined at representative spot frequency points.

Freq. (Hz)	M10		M20		M30		M40		M50	
	ε′	σ′	ε′	σ′	ε′	σ′	ε′	σ′	ε′	σ′
0.1	164.8	8.54E-11	14578.7	1.98E^− 8^	62,633	1.10E^− 7^	48,667	1.14E^− 7^	52,210	7.90E^− 8^
1.0	32.70	1.30E^− 10^	1309	2.23E^− 8^	3382	1.20 E^− 7^	2393	1.25E^− 7^	4242	8.47E^− 8^
10.0	12.46	1.67E^− 10^	155.5	2.36E^− 8^	347.5	1.24 E^− 7^	241.61	1.28E^− 7^	530	8.87E^− 8^
100.0	6.35	3.37E^− 10^	43.04	2.56E^− 8^	80.09	1.31 E^− 7^	73.18	1.33 E^− 7^	109	9.54E^− 8^
1000.0	2.83	8.13E^− 10^	15.89	3.12E^− 8^	27.07	1.41 E^− 7^	37.23	1.44E^− 7^	37.54	1.08E^− 7^
10000.0	2.27	1.97E^− 9^	7.63	4.74E^− 8^	14.49	1.74 E^− 7^	19.59	1.93E^− 7^	16.41	1.53E^− 7^
100000.0	2.03	7.07E^− 9^	4.60	1.04E^− 7^	8.02	3.07 E^− 7^	10.23	3.71E^− 7^	8.13	2.97E^− 7^
1000000.0	1.97	1.38E^− 8^	3.76	2.09E^− 7^	1.34	7.45 E^− 7^	7.23	9.40 E^− 7^	5.66	7.25E^− 7^

The permittivity, ε’, was evaluated at a fixed frequency of 10^3^ Hz as a representative frequency to thoroughly understand how the ceramic composition influences permittivity at different compositions (10%, 20%, 30%, 40%, and 50% of mullite). The findings indicate that M10 exhibitsthe lowest ε’ value (2.835). As the mullite concentration increased from M20 to M50, the ε’ value increases from 15.887 to approximately 37.225, as illustrated in Fig. 10b.

The electrical conductivity, σ(ω), of a material is an important parameter for analyzing its electrical performance, reflecting its ability to conduct electricity. A notable rise in conductivity is usually a result of higher concentration density and/or improved mobility of free-charge carriers.The aC conductivity σ_ac_ (ω) (usually called the real part of conductivity function σ′) related to the dielectric loss by^30^:

σ_ac_ = ε_o_ωε’’.

where ω is the angular frequency, ε_0_ is the free space permittivity (ε_0_ = 8.854 × 10^–12^ V^–1^ m^–1^), and ε ′′ (ω) is the imaginary part for permittivity. σ_ac_ (ω) arises from movements of free charge carriers towards the electric field.

**Fig. 11:**
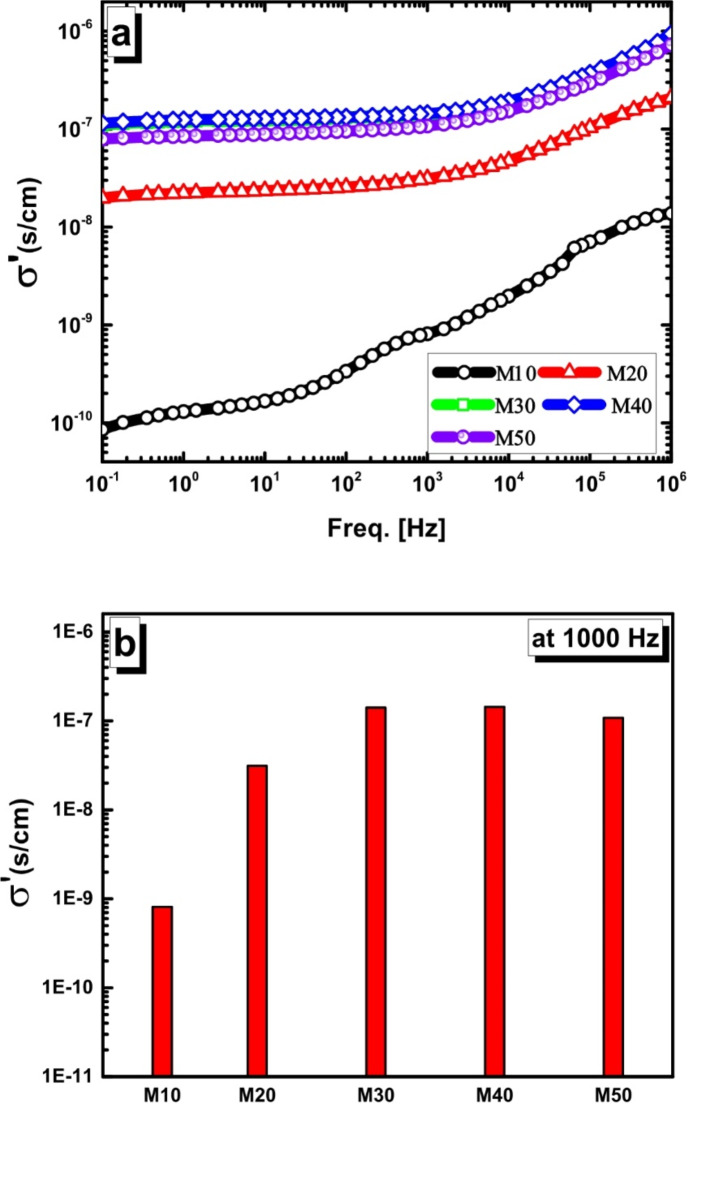
(**a**) Frequency dependence of Ac conductivity (*σ*_ac_) of M10, M20, M30, M40, and M50 compositions, and (**b**) *σ*_ac_ at a representative frequency f = 10^3^ Hz.

According to Fig. 11 and the list of selected frequencies in Table 6, the current study examined σ_ac_ (ω) throughout a broad frequency range. It can be noticed from the figure that σ_ac_ (ω) increases by two averages with increasing frequency. At frequencies below 10^2^ Hz, σ_ac_ (ω) grows very slowly, exhibiting a semi-plateau-like behavior. One possible explanation for this phenomenon is the influence of frequency-independent conductivity (σ_dc_). Conversely, σ_ac_ (ω) shows a notable rise in the 102–106 Hz frequency range, indicating frequency-dependent conductivity. The conductivity increased threefold in the low- frequency and about twofold in the high-frequency regions when the concentration of mullite was raised from M10 to M50. This suggests that an increase in the mobility and/or concentration of free charge carriers may lead to AC conductivity. As shown in Fig. 11b, the conductivity of all samples at a specific frequency of 10^3^ Hz was investigated as a representative study to understand the effect of mullite concentration on conductivity behavior .

**Fig. 12:**
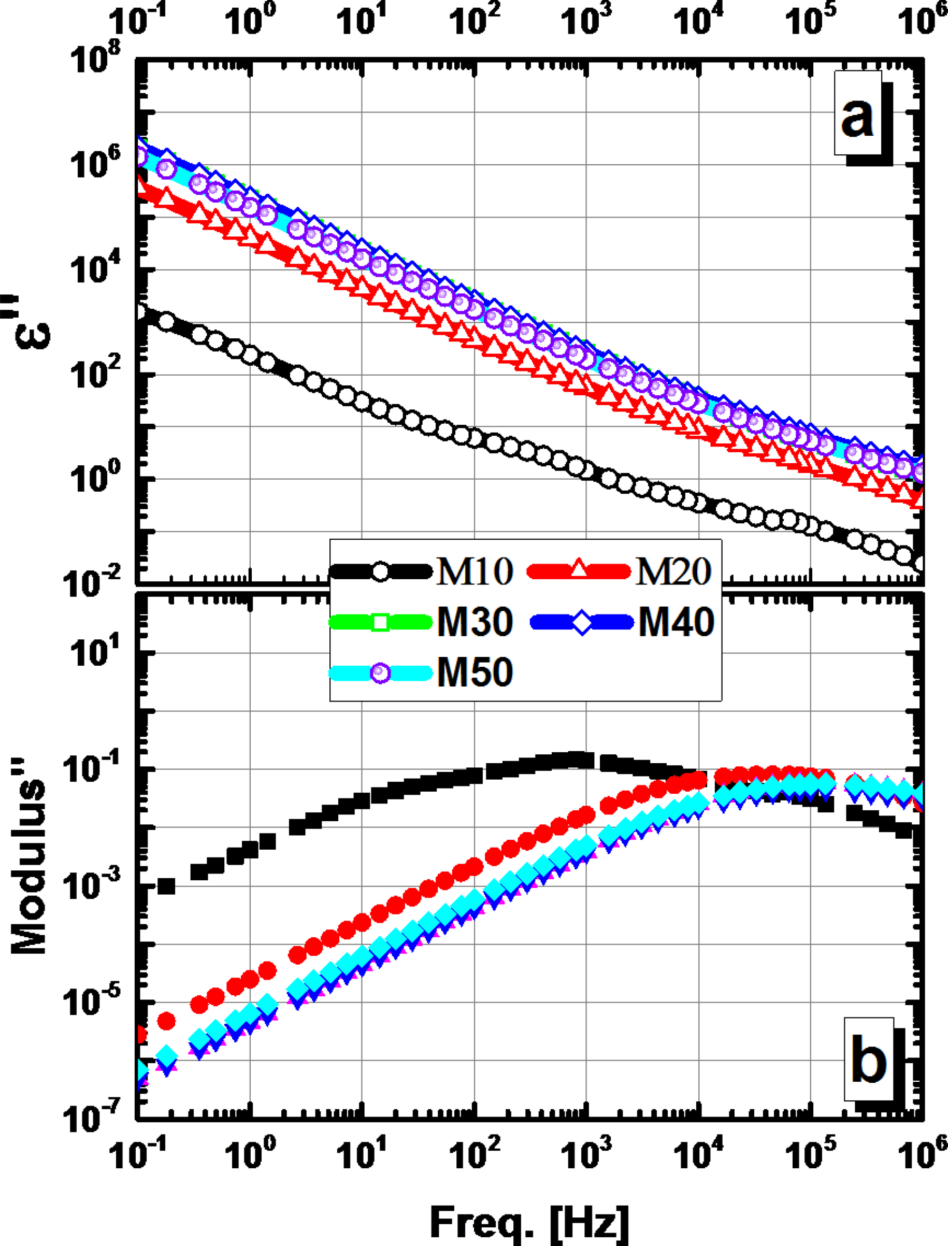
Frequency dependence of (**a**) dielectric loss (*ε’’*) and (**b**) Modulus’’ of the M10, M20, M30, M40, and M50 compositions.

In this study, we examined two types of losses in dielectric materials: dielectric loss (ε”) and electric modulus loss (M”). These losses occur when electric energy is dissipated as heat and/or leakage current. Figure 12 shows how these losses vary with frequency. Dielectric loss (ε”) decreases sharply as the frequency increases for all compositions. Dielectric loss occurs at low frequencies due to charge carrier immigration, ion hopping, and ion or dipole polarization in the material. However, the dipoles freeze at high frequencies, and the only source of dielectric loss is ion vibration. As a result, dielectric loss decreases as frequency increases^48^. On the other hand, the electric modulus loss (M”) increases as the concentration of mullite increases from M10 to M20, but shows no significant change when mullite concentration increases further from M20 to M50. This suggests that the influence of charge transport is overshadowing any contribution from polarization dynamics.

The complex electric modulus (M*) is a useful tool for analyzing the electrical response of compounds and investigating their dielectric relaxation processes. It can be calculated from the dielectric permittivity (ε*) using the following formula^30^:

M* = 1/ε*.

M* = M′ + jM′′.

Figure 12b illustrates the behavior of the imaginative part of the modulus (M”) as a function of frequency at room temperature. A single relaxation peak is observed in the imaginary part of the modulus (M”), which shifts to higher frequencies with increasing mullite concentrations. This shift indicates a decrease in hopping time, leading to faster dynamics of free charge carriers and a consequent conductivity enhancement. Therefore, the charge transport is dependent on both frequency and concentration. The shift in the peak position of the imaginary part of the composite modulus can be attributed to the observed variations in microstructure.

## Conclusion

- Industrial and mining wastes have been successfully used to produce refractory crystalline phases, such as cordierite, mullite, corundum, and spinel.
- The highest amount of recycled material used was in M50 which uses 72% recycled roller waste.
- Bulk density increases with higher mullite and lower cordierite content. It also increases with rising temperature. Conversely, porosity and water absorption decrease with an increase in both mullite content and temperature.
- It was found that increasing the percentage of mullite enhances the bending strength while density, grain size, and porosity decrease.
- The microstructure develops finer grains with increasing mullite content and density.
- The study showed that the electrical insulation properties improved by increasing the percentage of mullite from M10 to M50, indicating that these materials have significant potential for various electrical applications.
- This approach successfully protects protects the environment by converting these harmful wastes, which occupy a large area of land, into refractory ceramics.

## Data Availability

The data that support the findings of this study are available from the corresponding author [G.A.K] upon reasonable request.
